# Emergency medical admissions and COVID-19: impact on 30-day mortality and hospital length of stay

**DOI:** 10.1007/s11845-021-02752-7

**Published:** 2021-08-30

**Authors:** Richard P. Conway, Declan G. Byrne, Deirdre M. R. O’Riordan, Brian D. Kent, Barry M. J. Kennedy, Clíona M. Ní Cheallaigh, Brian P. O’Connell, Nadim B. Akasheh, Joseph G. Browne, Bernard M. Silke

**Affiliations:** Department of Internal Medicine, St. James’s Hospital, Dublin 8, Ireland

**Keywords:** COVID-19, Emergency medical admissions, Mortality, Resource utilization

## Abstract

**Background:**

The COVID-19 pandemic has put considerable strain on healthcare systems.

**Aim:**

To investigate the effect of the COVID-19 pandemic on 30-day in-hospital mortality, length of stay (LOS) and resource utilization in acute medical care.

**Methods:**

We compared emergency medical admissions to a single secondary care centre during 2020 to the preceding 18 years (2002–2019). We investigated 30-day in-hospital mortality with a multiple variable logistic regression model. Utilization of procedures/services was related to LOS with zero truncated Poisson regression.

**Results:**

There were 132,715 admissions in 67,185 patients over the 19-year study. There was a linear reduction in 30-day in-hospital mortality over time; over the most recent 5 years (2016–2020), there was a relative risk reduction of 36%, from 7.9 to 4.3% with a number needed to treat of 27.7. Emergency medical admissions increased 18.8% to 10,452 in 2020 with COVID-19 admissions representing 3.5%. 18.6% of COVID-19 cases required ICU admission with a median stay of 10.1 days (IQR 3.8, 16.0). COVID-19 was a significant univariate predictor of 30-day in-hospital mortality, 18.5% (95%CI: 13.9, 23.1) vs. 3.0% (95%CI: 2.7, 3.4)—OR 7.3 (95%CI: 5.3, 10.1). ICU admission was the dominant outcome predictor—OR 12.4 (95%CI: 7.7, 20.1). COVID-19 mortality in the last third of 2020 improved—OR 0.64 (95%CI: 0.47, 0.86). Hospital LOS and resource utilization were increased.

**Conclusion:**

A diagnosis of COVID-19 was associated with significantly increased mortality and LOS but represented only 3.5% of admissions and did not attenuate the established temporal decline in overall in-hospital mortality.

## Introduction

Coronavirus disease 2019 (COVID-19) has caused high rates of critical illness and mortality [1, 2] with burdens placed on national healthcare systems. Case fatality rates for COVID-19 have varied dramatically worldwide, perhaps because of differences in populations, public health, and healthcare systems [3]; it appears also that outcomes may have improved over time [4]. In acute medicine, the COVID-19 pandemic led to a major reorganization of in-hospital care with substantial restrictions, either provider-imposed or consumer reactive, on non-COVID-19 attendance and admissions [5]. Staffing rotas have been reconfigured, with attempted higher levels of consultant presence to anticipate the effect of radical reorganization of care on admission and discharge patterns [5].

Much attention has been centred on the question of underlying comorbidity and its impact; meta-analysis has suggested that specific conditions such as cardiovascular disease predispose to worse outcomes [6]. The importance of illness severity at presentation has not been reported previously [7]. In this paper, we examine the impact of the first year of the COVID-19 epidemic on the demographics and outcomes of emergency medical admissions overall, and specifically explore factors associated with outcomes in those with a diagnosis of COVID-19.

## Methods

### Background

St. James’s Hospital, Dublin, serves as a secondary care centre for emergency admissions in a catchment area with a population of 270,000 adults. St. James is based in an inner city area with an ageing population and high intrinsic rates of deprivation and comorbidity [7, 8]. All emergency medical admissions are admitted from the emergency department (ED) to an acute medical admission unit (AMAU), the operation and outcome of which have been described elsewhere [9–12]. There were changes to this model of care during the COVID-19 pandemic, primarily with COVID-19 patients being admitted to designated wards; non-COVID-19 patients continued to follow the above model. The current study evaluates all emergency medical admissions between 2002 and 2020.

### Data collection

An anonymous patient database was employed, assembling core information from each clinical admission including details from the patient administration system, the national hospital in-patient enquiry (HIPE) scheme, the patient electronic record, and the laboratory data. HIPE is a national database of coded discharge summaries from acute public hospitals in Ireland [13]. The International Classification of Diseases, Ninth Revision, Clinical Modification (ICD-9-CM) has been used for both diagnosis and procedure coding from 1990 to 2005 and ICD-10-CM since then. Data included parameters such as the unique hospital number, admitting consultant, date of birth, gender, area of residence, principal and up to nine additional secondary diagnoses, principal and up to nine additional secondary procedures, and admission and discharge dates. Additional information cross-linked and automatically uploaded to the database includes physiological, haematological, and biochemical parameters.

### Risk predictors

Derangement of admission biochemical parameters may be utilized to predict clinical outcome. We have previously derived and applied an Acute Illness Severity Score (AISS) [14], predicting 30-day in-hospital mortality from parameters recorded in the ED. A weighted age adjusted score was derived; six risk groups (I–VI) were identified with cut-points for 30-day in-hospital mortality set at 1, 2, 4, 8, and 16%. We further adjusted for comorbidity as described below. In addition, categories of (1) no blood culture request, (2) negative blood culture, and (3) positive blood culture were identified and used as an adjustor in the multivariable logistic regression model [15].

### Comorbidity score

Patient morbidity was assessed by a comorbidity score [7]. To devise the score, we searched ICD Codes that captured chronic physical or mental health disorders that limit people in activities that they generally would be expected to be able to perform were grouped according to the following ten systems: (i) cardiovascular, (ii) respiratory, (iii) neurological, (iv) gastrointestinal, (v) diabetes, (vi) renal, (vii) neoplastic disease, (viii) others (including rheumatological disabilities), (ix) ventilatory assistance required, and (x) transfusion requirement. In addition, we searched our hospital’s other databases for evidence of diabetes (Diamond database) [16], respiratory insufficiency (FEV1 < 2 L), troponin status (high sensitivity troponin ≥ 25 ng/L) [17], low albumin (< 35 G/dL), and anaemia (haemoglobin levels < 10 G/dL) or chronic renal insufficiency—MDRD < 60 mL/min * 1.73 m^2^ [18]. Each component of the score was then weighted according to 30-day in-hospital mortality.

### Statistical methods

Descriptive statistics were calculated for background demographic data, including means/standard deviations (SD), medians/inter-quartile ranges (IQR), or percentages. Comparisons between categorical variables and mortality were made using chi-square tests. We adjusted the outcome computation (30-day in-hospital mortality) for other known predictor variables including AISS [14], comorbidity score [7], and blood culture results [15]. Thirty-day in-hospital mortality is defined as a death occurring for any reason in the hospital within 30 days of the admission date. We employed a logistic model with robust estimate to allow for clustering; the correlation matrix thereby reflected the average discrete risk attributable to each of these predictor variables [14]. Of course, over a prolonged observation period of 19 years, many patients were admitted more than once. For example, those admitted more than one, two, or three times were 49.5%, 31.8%, and 22.4%, respectively, with 5.4% admitted > 10 times each. Clearly, there will be a difference in mortality rates if calculated by admission or by patient (only last admission considered if > 1); we specifically state the calculation basis throughout.

Logistic regression analysis identified potential mortality predictors and then tested those that proved to be significant univariate predictors (*p* < 0.1 by Wald test) to ensure that the model included all variables with predictive power. We used the margin command in Stata to estimate and interpret adjusted subgroup predictions controlling for other variables, using computations of average marginal effects. Margins are statistics calculated from predictions of a previously fitted model at fixed values of some covariates and averaging or otherwise over the remaining covariates.

We were interested to determine whether COVID-19 status, due to case complexity and an extended hospital stay, might be associated with additional investigations. We quantitated in-hospital procedures during each admission, including allied service utilization (physiotherapy, speech and language, dietetics, social, occupational therapy, and psychiatric attendance) and interventions including ventilation, bronchoscopy, GI endoscopy/colonoscopy, and coronary angiography. We regressed the sum of these against hospital length of stay (LOS), using truncated Poisson regression, by COVID-19 status. The dependent variable of LOS is restricted to certain values; the predictor variables were therefore regressed against LOS using the truncated Poisson model. We used robust standard errors for the parameter estimates, as recommended by Cameron and Trivedi [19]. The Poisson regression coefficients are the log of the rate ratio: one can interpret the coefficients in terms of incidence rate ratios (IRR).

Adjusted odds ratios (OR) and 95% confidence intervals (CI) were calculated for those predictors that significantly entered the model (*p* < 0.10). Statistical significance at *p* < 0.05 was assumed throughout. Stata v.16 (Stata Corporation, College Station, TX) statistical software was used for analysis.

## Results

### Patient demographics

There were a total of 132,715 emergency medical admissions in 67,185 patients over the 19-year study period (2002–2020). Total emergency medical hospital admissions increased from 5476 in 2002 to 8485 in 2019 (+ 54.9%) with a further increase in 2020 to 10,452 (+ 18.8% increase vs. 2019). However, COVID-19 cases only represented 3.5% of admissions in 2020 (*n* = 361; Table 1). The total number of COVID-19 hospital admissions in Ireland in 2020 was 5985 (https://www.gov.ie/en/press-release/166a4-statement-from-the-national-public-health-emergency-team-thursday-31-december/). For all admissions, males represented 50.8%. The median (IQR) age was 63.1 (43.7, 77.6) years, with the upper 10% boundary at 85.3 years. The median (IQR) LOS was 4.9 (2.0, 9.4) days. COVID-19 cases were older at a median of 66.6 years (51.7, 79.1) vs. 61.1 years (46.0, 74.4) and had a longer median LOS of 11.2 days (5.2, 26.6) vs. 3.9 days (1.5, 8.6). Total in-hospital mortality was greater for COVID-19 cases at 17.4% vs. other admissions at 3.3%. COVID-19 cases tended to have greater illness severity (AISS top three grades 75.2% vs. 68.5%) and comorbidity scores (> 10 points 10.6% vs. 2.7%) and more positive blood cultures (13.4% vs. 2.9%).

**Table 1:** Characteristics of emergency medical admissions by COVID-19 status

	Non-COVID-19	COVID-19	*p*-value
	(*N* = 10,091)	(*N* = 361)	
Age (years)			
Mean (SD)	59.7 (18.8)	64.1 (18.4)	< 0.001
Median (Q1, Q3)	61.1 (46.0, 74.4)	66.6 (51.7, 79.1)	
Length of stay (days)			
Mean (SD)	7.9 (13.49)	23.0 (33.1)	< 0.001
Median (Q1, Q3)	3.9 (1.5, 8.6)	11.2 (5.2, 26.6)	
Gender			
Male	5498 (54.6%)	192 (53.8%)	0.78
Female	4568 (45.4%)	165 (46.2%)	
Hospital mortality			
Alive	9734 (96.7%)	295 (82.6%)	< 0.001
Dead	332 (3.3%)	62 (17.4%)	
Acute Illness Severity Score			
1	486 (5.1%)	20 (5.8%)	< 0.001
2	985 (10.3%)	28 (8.1%)	
3	1544 (16.2%)	38 (11.0%)	
4	1789 (18.7%)	57 (16.5%)	
5	1658 (17.4%)	53 (15.3%)	
6	3094 (32.4%)	150 (43.4%)	
Comorbidity score			
< 6	7396 (73.5%)	171 (47.9%)	< 0.001
6	2404 (23.9%)	148 (41.5%)	
10	229 (2.3%)	33 (9.2%)	
13	37 (0.4%)	5 (1.4%)	
Charlson index			
0	4422 (55.5%)	189 (58.9%)	0.14
1	2076 (26.1%)	87 (27.1%)	
2	1465 (18.4%)	45 (14.0%)	
Blood culture group			
1	8315 (82.6%)	115 (32.2%)	< 0.001
2	1455 (14.5%)	194 (54.3%)	
3	296 (2.9%)	48 (13.4%)	

### Effect on 30-day hospital mortality over time

**Fig. 1:**
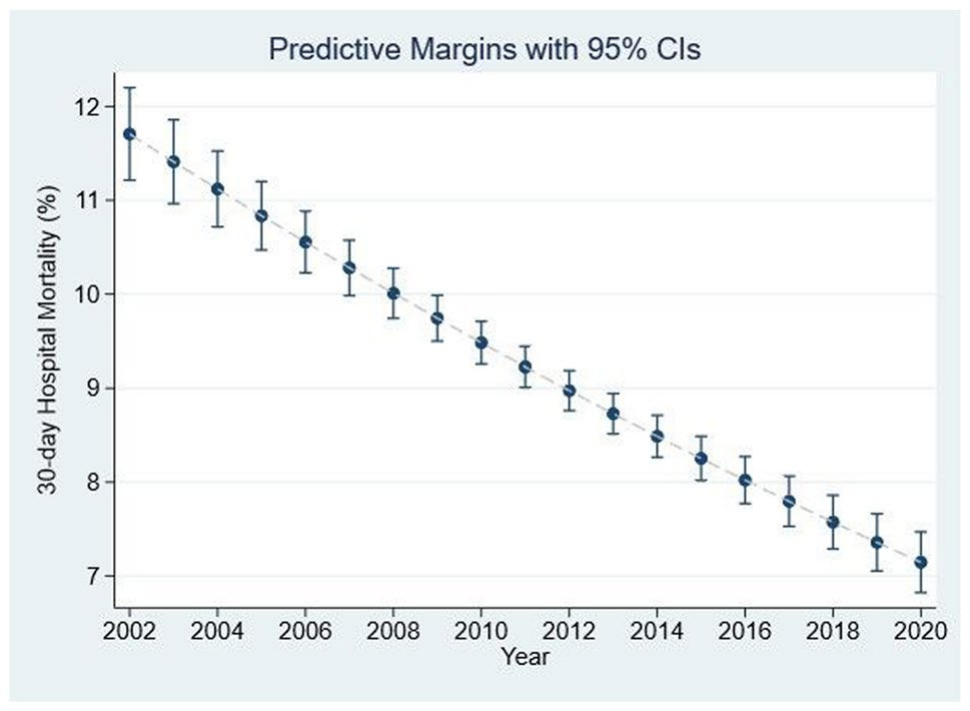
Time impact (2002–2020) on 30-day in-hospital per patient mortality. Overall mortality fell by 66% with NNT of 12

The long-term trend of reduction in 30-day in-hospital mortality, year on year, was uninterrupted by COVID-19 admissions. However, COVID-19 cases only represented 3.5% of the 10,452 admissions in 2020; the higher mortality with a COVID-19 admission was insufficient to impact the linear decrease in 30-day in-hospital mortality over time (Fig. 1). As calculated per patient, the 30-day in-hospital mortality over the 19-year period averaged at 8.7% (95%CI 8.5 to 8.9%); there was a relative risk reduction (RRR) of 66% between 2002 and 2020, from 12.7 to 4.3% (*p* = 0.001) with a NNT (number needed to treat) of 12. The mortality improvement continued in 2020, and with respect to the 5-year period of 2016–2020, it showed a RRR of 36% from 7.9%, with a NNT of 27.7.

### COVID-19 and comorbidity profile relationship to 30-day in-hospital mortality

**Fig. 2:**
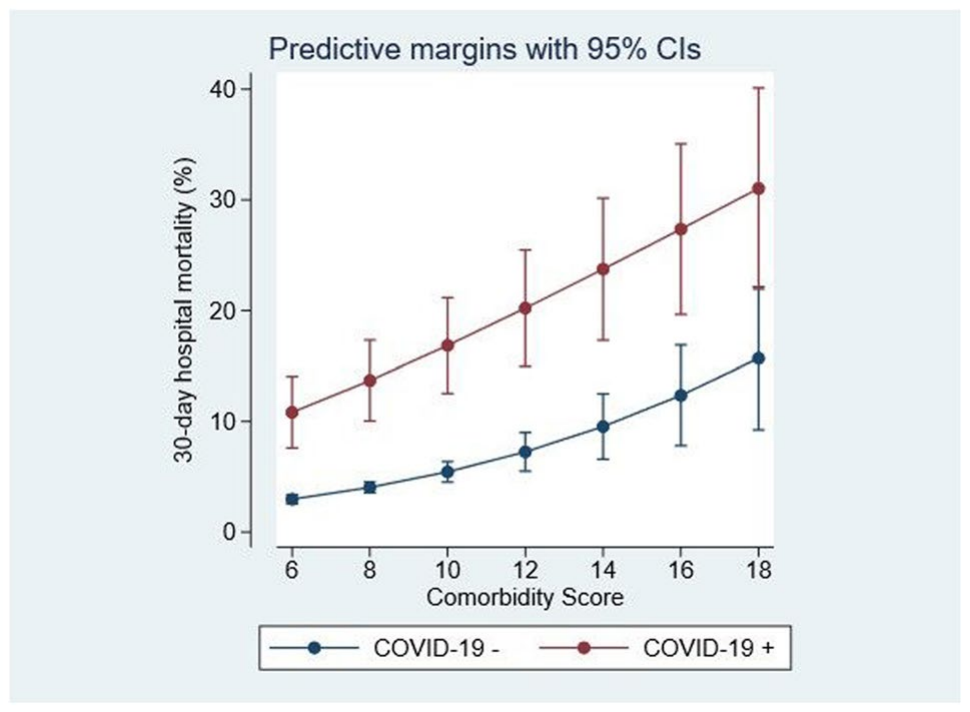
COVID-19 status and impact on 30-day in-hospital episode mortality. Mortality at any comorbidity score increased at 6, 10, and 14 points form 3.0%, 5.4%, and 9.5% to COVID-19 of 10.8%, 16.8%, and 23.7%

COVID-19 status was a significant univariate predictor of 30-day in-hospital mortality—OR 7.3 (95%CI: 5.3, 10.1) (Fig. 2). Overall, 18.6% (67/361) of COVID-19 admissions required an ICU admission; their median ICU stay was 10.1 days (IQR 3.8, 16.0). The 30-day hospital mortality for COVID-19 admissions was 18.5% (95%CI: 13.9, 23.1), compared with 3.0% (95%CI: 2.7, 3.4) for non-COVID-19 cases. COVID-19 status after adjustment for other predictors had wide confidence limits OR 1.99 (95%CI: 0.6, 6.6) and was not significant (*p* = 0.26). Important factors in predicting an in-hospital death by day 30 were ICU admission OR 12.4 (95%CI: 7.7, 20.1) and AISS, comorbidity score, Charlson comorbidity index, and blood culture results (Table 2). In the multiple variable model, being older (> = 70 years) tended to have worse outcomes (*p* = 0.09)—OR 1.36 (95%CI: 0.95, 1.93). Mortality for COVID-19 admissions improved over time; the mid-year (180 case split) model adjusted mortality reduction was borderline (*p* = 0.06)—OR 0.74 (95%CI: 0.55, 1.01), but for the last third of 2020, COVID-19 admission (249/112 case split) outcomes improved—OR 0.64 (95%CI: 0.47, 0.86) (*p* = 0.003).

**Table 2:** Multiple variable logistic regression prediction by COVID-19 status

Variable	Odds ratio	Std. err	*z*	*p* >|*z*|	95% conf. interval	
COVID-19 status	1.99	1.21	1.1	0.26	0.60	6.55
ICU admission	12.44	3.04	10.3	0.00	7.71	20.10
Age > 70 years	1.36	0.24	1.7	0.09	0.95	1.93
Morbidity score	1.22	0.04	6.3	0.00	1.15	1.30
Illness severity	3.84	0.62	8.3	0.00	2.80	5.27
Charlson index	1.45	0.13	4.1	0.00	1.21	1.73
Blood culture	1.77	0.21	4.8	0.00	1.40	2.24
COVID older^##^	5.78	3.68	2.8	0.01	1.66	20.17
COVID ICU^##^	0.05	0.03	− 4.7	0.00	0.02	0.18

^##^Significant interactions in multiple-variable model

### LOS and COVID-19 status

**Fig. 3:**
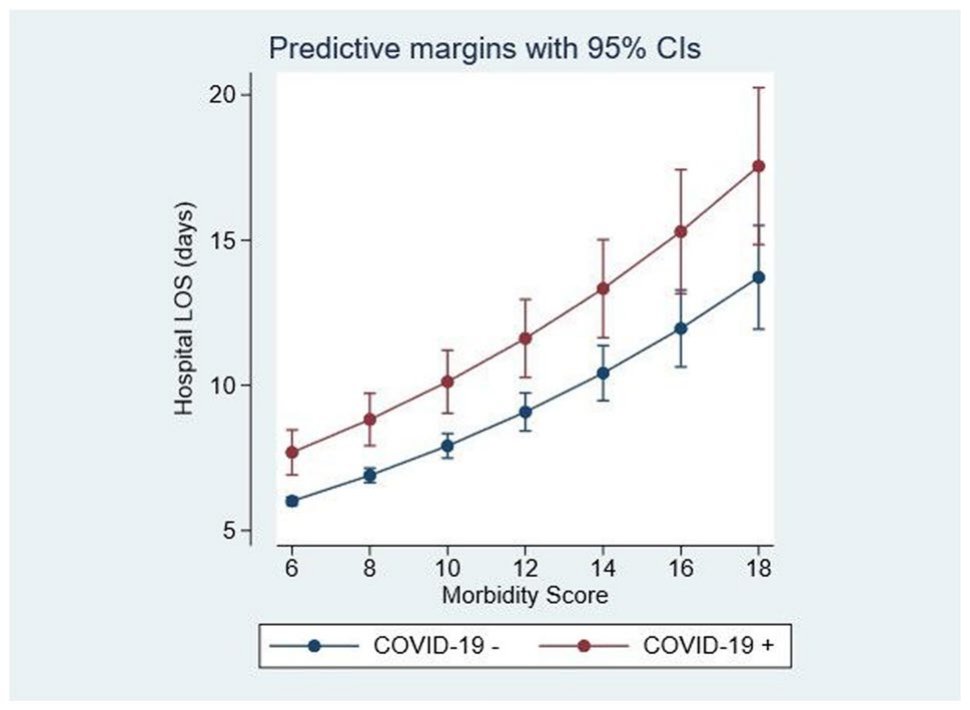
COVID-19 status and hospital length of stay (LOS). The hospital LOS showed comorbidity dependence but was significantly higher for COVID-19 admissions. The comorbidity score was < 6 points in 66.5%, between 6 and 10 in 29.3% with only 3.8% scored at > 10 points

LOS was considerably extended for COVID-19 admissions (Fig. 3). COVID-19-positive admissions were, after adjustment for case complexity (AISS, comorbidity score, Charlson index, blood culture results), more likely to have an extended LOS—IRR 1.43 (95%CI: 1.32, 1.56) (*p* = 0.001).

### COVID-19 status and resource utilization

**Fig. 4:**
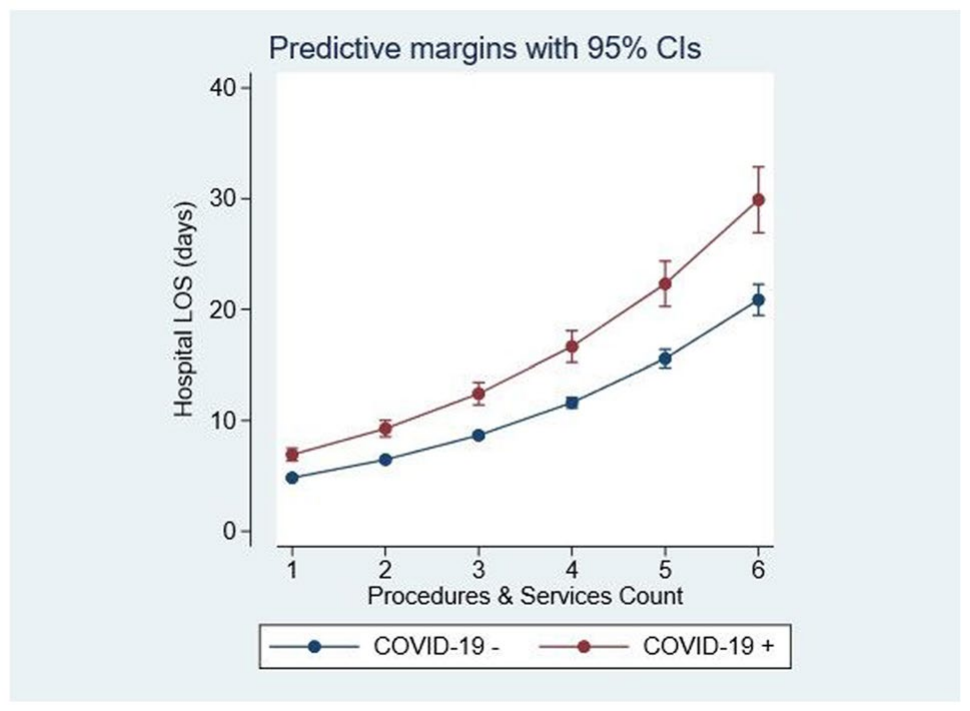
Hospital LOS and resource utilization related to COVID-19 status, derived from the zero truncated Poisson regression model. The hospital LOS and utilization of service/procedure were linearly related; however, COVID-19 status was associated with greater utilization of resources

There was a linear relationship between the hospital LOS and the number of procedures/interventions, when unadjusted for case complexity: IRR 1.34 (95%CI: 1.32, 1.36) (*p* < 0.001) (Fig. 4).

## Discussion

We have demonstrated several important findings in this single-centre study. COVID-19 admissions represented 3.5% of admissions in 2020. Contrary to the expressed view that non-COVID admissions would decrease during the pandemic due to patients avoiding the ED, we have demonstrated an increase in non-COVID emergency medical admissions in the same time frame. COVID-19 admissions were associated with greatly increased 30-day in-hospital mortality and length of stay. Clinical mortality outcomes of COVID-19 admissions improved significantly over time, with mortality decreasing by 36% later in 2020; this may relate to both greater experience in treating COVID-19 and the clinical implementation of the proven benefit of glucocorticoids in COVID-19 [20, 21].

There is a view that COVID-19 patients have a high mortality due to underlying comorbidity such as COPD, cardiovascular or cerebrovascular disease, and diabetes [6]. Our data includes a measure of the admission illness severity [14], predicting 30-day in-hospital mortality from parameters recorded in the ED [22]. This is a weighted measure of the disturbance in biochemistry and reflects the extent of the failure of homeostatic compensation. It is a not routine parameter available as a model adjustor, unlike comorbidity which is generally derived from ICD Codes such as the Charlson comorbidity index [23], when modelling and relating to mortality outcomes. If it were the case that comorbidity is a substantial determinant of mortality outcomes, then one would anticipate curves close together a low morbidity, with separation as the comorbidity burden increased; our data (Fig. 1) showed no such trend with parallel curves. Overall, COVID-19 cases had worse outcomes than non-COVID-19 admissions; the adjusted multiple variable model prediction for 30-day hospital mortality was 11.4% vs. 3.6%—a 3.2-fold increase after adjustment for case complexity and comorbidity. However, although major predictors of comorbidity, illness severity, and blood culture results were all individually predictive, the interaction function indicated that the independent underlying predictor was illness severity (high AISS); AISS was the only predictor remaining significant in the full model, including prediction interactions.

The overall mortality rate cited in earlier series of hospitalized patients with COVID-19 was 11–15%, but the later experience suggested a fall to 2–3% [24]; these figures are very far from our data. Of course, comparing death rates across populations is problematic with social determinants of health contributed to differential mortality rates. In Brazil, older persons > 70 years had a greater than 55% mortality that fell to 12% in patients younger than 40 years [25]. However, some very large temporal mortality reductions in older UK adults across the early pandemic were suggested, with more than a 50% decrement in patients older than 80 years [26]; Canadian experience suggests a substantial fall month on month, from 25.6 to 7.6% [4]. Other UK experience has suggested an 11·8% reduction in adjusted 28-day inpatient mortality over time for critically ill patients [27]. We would feel that a model-adjusted fall of the order of 30% would be substantial and consistent with reasonable expectations; a model outcome prediction tool for COVID-19 with laboratory elements appears to have similar predictive mortality rates related to a similar range of computed scores to our data [28].

In terms of resource utilization, adjusted for case complexity and comorbidity, COVID-19 admissions had a more extended hospital episode (+ 26%) with increased demand on hospital procedures and services (+ 34%). We have shown a significant increase in non-COVID emergency medical admissions in 2020, in contrast to reports from elsewhere [29, 30]. Crisis planning resulting in the recruitment of more participating consultants and specialities into the admitting acute medicine service in our institution may have adjusted upwards somewhat the numbers of patients classified as emergency medical admissions, but a + 22.8% increase vs. 2019 in volume with the added burden of COVID-19 appears a very large additionality for the acute medicine service. It is possible that limitations in access to scheduled care services resulted in more emergency medical admissions as this was sometimes the only route of care access remaining functional during the pandemic. It will be interesting to see whether this augmentation in admission volume represents a transient phenomenon.

There are some limitations to our current analysis. Our study was conducted in a single large academic teaching hospital serving an urban area with a population with high deprivation; our results require replication in other centres and settings to confirm the external validity of our findings [8, 31, 32]. We have adjusted for a large number of potential outcome predictors in our multiple variable models: however, it is possible that residual unmeasured confounders remain. There may have been overfitting of our models; however, the large numbers of cases included mitigates against this. It is impossible to fully remove the effects of COVID-19 from the dramatic restructuring of care which happened due to the crisis management of the situation and this may have had unpredictable additional effects on outcomes [32]. Our database collects extensive information on all medical admissions; however, certain variables which have been shown to be risk factors for poor COVID outcomes, including BMI and ethnicity, are not included. Our study included data until the end of 2020, encompassing the first two waves of COVID-19 in Ireland; it is possible that the third wave of COVID-19 in early 2021 may have had a different impact and this should be assessed in future studies.

In conclusion, we have demonstrated that COVID-19 admissions have a significantly increased mortality and hospital length of stay. Outcomes in patients with COVID-19 appear to be mediated by illness severity rather than comorbidity or other predictors.

## Data Availability

No further data is available for sharing. All pertinent data have been published in this manuscript.
